# Blunted brain responses to neutral faces in healthy first-degree relatives of patients with schizophrenia: an image-based fMRI meta-analysis

**DOI:** 10.1038/s41537-024-00452-6

**Published:** 2024-03-19

**Authors:** Anna M. Fiorito, Giuseppe Blasi, Jérôme Brunelin, Asadur Chowdury, Vaibhav A. Diwadkar, Vina M. Goghari, Ruben C. Gur, Jun Soo Kwon, Tiziana Quarto, Benjamin Rolland, Michael J. Spilka, Daniel H. Wolf, Je-Yeon Yun, Eric Fakra, Guillaume Sescousse

**Affiliations:** 1 Université Claude Bernard Lyon 1, CNRS, INSERM, Centre de Recherche en Neurosciences de Lyon CRNL U1028 UMR5292, PSYR2, 69500 Bron, France; 2 Centre Hospitalier Le Vinatier, Bron, France; 3 Group of Psychiatric Neuroscience, Department of Translational Biomedicine and Neuroscience, University of Bari Aldo Moro, Bari, Italy; 4 Azienda Ospedaliero-Universitaria Consorziale Policlinico, Bari, Italy; 5 Department of Psychiatry & Behavioral Neurosciences, Wayne State University, Detroit, MI USA; 6 Department of Psychological Clinical Science, University of Toronto, Toronto, ON Canada; 7 Department of Psychiatry, Perelman School of Medicine, University of Pennsylvania, Philadelphia, PA USA; 8 Department of Psychiatry, Seoul National University College of Medicine, Seoul, Korea; 9 Department of Humanities, University of Foggia, Foggia, Italy; 10 Winterlight Labs, Toronto, ON Canada; 11 Department of Psychiatry, University Hospital of Saint-Etienne, Saint-Etienne, France

**Keywords:** Neuroscience, Schizophrenia

## Abstract

Schizophrenia is characterized by the misattribution of emotional significance to neutral faces, accompanied by overactivations of the limbic system. To understand the disorder’s genetic and environmental contributors, investigating healthy first-degree relatives is crucial. However, inconsistent findings exist regarding their ability to recognize neutral faces, with limited research exploring the cerebral correlates of neutral face processing in this population. Thus, we here investigated brain responses to neutral face processing in healthy first-degree relatives through an image-based meta-analysis of functional magnetic resonance imaging studies. We included unthresholded group-level T-maps from 5 studies comprising a total of 120 first-degree relatives and 150 healthy controls. In sensitivity analyses, we ran a combined image- and coordinate-based meta-analysis including 7 studies (157 first-degree relatives, 207 healthy controls) aiming at testing the robustness of the results in a larger sample of studies. Our findings revealed a pattern of decreased brain responses to neutral faces in relatives compared with healthy controls, particularly in limbic areas such as the bilateral amygdala, hippocampus, and insula. The same pattern was observed in sensitivity analyses. These results contrast with the overactivations observed in patients, potentially suggesting that this trait could serve as a protective factor in healthy relatives. However, further research is necessary to test this hypothesis.

## Introduction

Schizophrenia is a debilitating psychiatric disorder affecting approximately 1% of the population. According to the aberrant salience hypothesis^1^, patients with schizophrenia tend to attribute motivational significance to stimuli that are considered as irrelevant by healthy subjects. Consistent with this theory, patients with schizophrenia exhibit heightened subjective arousal in response to emotionally neutral stimuli including faces, scenes, words, sounds^2^. Notably, studies have reported that patients misattribute emotional significance to neutral faces, and tend to perceive such faces as negatively valenced^3–5^. This biased interpretation of faces, a central element of our social environment, significantly impacts patients’ social skills and overall functioning^6^.

At the brain level, a recent functional magnetic resonance imaging (fMRI) meta-analysis by Dugré and colleagues^7^ examined the processing of emotionally neutral stimuli in schizophrenia. Compared with healthy controls, patients with schizophrenia showed increased activation in limbic regions in response to neutral stimuli, in particular when processing neutral faces. This finding supports the idea that patients do not perceive emotionally neutral faces as being devoid of emotional significance.

Although substantial progress has been made in understanding the processing of emotionally neutral stimuli in schizophrenia, there is a dearth of research examining this phenomenon in healthy first-degree relatives of patients with schizophrenia. Indeed, these individuals share on average 50% of their genes with their siblings diagnosed with schizophrenia and are considered to be at familial risk. Since many of these genes are involved in brain development, genetic variations are likely to impact brain structure and ultimately brain function. It is thus to be expected that common genetic heritability may manifest at the neuroimaging level^8–10^. Studies focusing on first-degree relatives are thus crucial, as they are unaffected by potential confounding factors (such as chronic effects of the disease, medication treatment, or physiological adaptation secondary to the illness) which are inextricably tied to schizophrenia itself, thus limiting our understanding of the cerebral correlates of this chronic illness.

Some previous work in first-degree relatives has reported a tendency to over-attribute negative emotions to neutral faces, in line with the behavioral bias observed in patients^11,12^. However, these findings have not been replicated in more recent studies with larger sample sizes or confirmed by meta-analyses^13–16^. To date, neuroimaging studies have mainly focused on the general processing of emotional faces, with recent meta-analytical work showing that brain activity patterns in individuals at enhanced risk are similar to healthy controls^17^. Only a few studies have investigated the neural mechanisms associated with the processing of neutral faces in first-degree relatives. This scarcity of studies arises partly from the challenges inherent to conducting research within this population. As a consequence, the few studies that have tackled this question have typically relied on relatively modest sample sizes, hence providing limited statistical power^18,19^. In this context, the main objective of our study was to rely on a meta-analytical approach to consolidate the results from small sample studies that have investigated brain responses to neutral faces in healthy first-degree relatives. We aimed to identify potential brain differences with healthy controls, eventually advocating for further exploration and investigation within this domain in forthcoming research.

Due to the scarcity of studies investigating this question, we systematically reviewed all task-based fMRI studies that used neutral faces as stimuli, regardless of whether activations to neutral faces were specifically investigated or even reported in the original article. Authors were contacted and asked to provide whole-brain maps showing 1) brain responses towards neutral faces as compared to a control condition separately in first-degree relatives and healthy controls (which were used to perform within-group meta-analyses), 2) brain responses towards neutral faces as compared with a control condition in first-degree relatives versus healthy controls (which were used to perform between-group meta-analyses).

## Materials and methods

This meta-analysis was conducted following the guidelines of the Preferred Reporting Items for Systematic Reviews and Meta-analyses (PRISMA^20^), using an approach similar to our recent work^17^. The protocol for this meta-analysis was not pre-registered. We conducted a comprehensive literature search using PubMed and Web of Science, identifying records published until August 22nd, 2023. The search used a combination of terms constructed according to 4 stems relating to (a) schizophrenia (schizophren*, psychosis), (b) first-degree relatives (relatives, first-degree, siblings, twins, brothers, sisters, offspring, parents, genetic risk) (c) neuroimaging (neuroimaging, functional magnetic resonance imaging, fMRI), and (d) face processing (emotion*, affect, mood, face, facial) (see Supplementary Methods for the exact combination of keywords employed in the search strategy). Additionally, we performed a reference list inspection of the included studies to identify articles that were not identified through the database search.

### Inclusion and exclusion criteria

Inclusion criteria were the following: studies that (a) were written in English; (b) reported a comparison between healthy first-degree relatives and healthy controls; (c) used task-based fMRI with face processing, and (d) used neutral faces.

It should be noted that this meta-analysis specifically focused on faces as neutral stimuli in order to increase homogeneity among the included contrasts and acknowledge the significant social importance associated with these stimuli, especially in light of previous findings in patients^7^. Here we conceptualized neutral face processing as the collective neural processes engaged in perceiving, recognizing, and interpreting faces without any salient visual features, and thereby assumed to lack emotional significance.

The following exclusion criteria were applied: studies that (a) did not report a neutral face versus control condition/baseline contrast, or for which the authors could not provide the contrast when queried; (b) did not use visual stimuli of faces in their protocol; (c) did not use original samples of individuals (i.e., used same data as other included study); (d) could only provide a general F-statistic instead of direct comparisons between groups; (e) were published conference papers, and (f) were systematic reviews or meta-analyses.

It should be noted that we systematically searched for studies whose methodology was *capable* of investigating neutral face processing, regardless of whether they actually reported the analysis of interest in the original article. This approach was adopted to maximize the pool of potentially eligible studies.

### Included studies

**Fig. 1:**
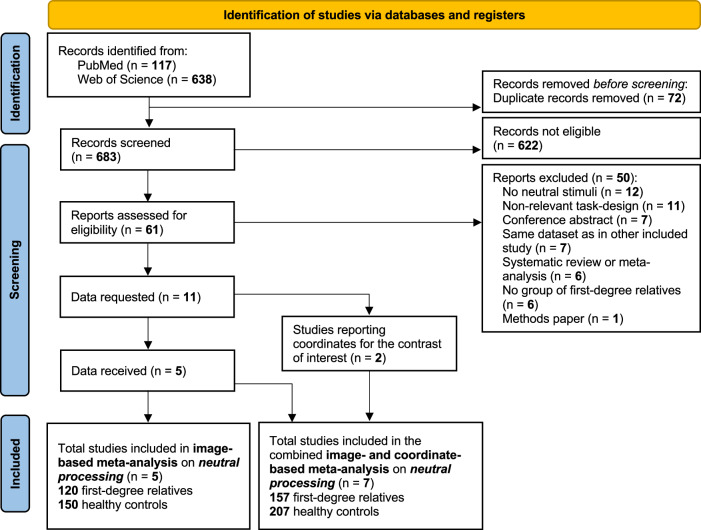
Flow chart. This diagram depict the selection procedure of studies.

The literature search yielded 683 unique papers (see Fig. 1). Initial screening based on title and abstract was performed by AMF. After these exclusions, 61 full articles were independently screened to be assessed for eligibility by AMF and GS. Disagreements were resolved through discussion and mediation by a third author (EF). The application of inclusion and exclusion criteria filtered the analysis to a total of 11 papers (see Supplementary Methods for details on study exclusion). The corresponding authors of these papers were contacted to obtain unthresholded group-level T-maps, associated with within- and between-groups effects. Following this correspondence, we received data from 5 studies that were included in the image-based meta-analysis.

Among the studies for which we could not retrieve data, 2 reported the peak coordinates of the contrast of interest and were thus included in a sensitivity analysis combining these coordinate-based results with the aforementioned image-based results (i.e., 7 studies in total). We were unable to include data from the remaining studies^21–24^ due to various reasons, including no reported peak coordinates for the contrast of interest, non-responsive corresponding authors (after multiple reminders), and unavailable or lost data. Furthermore, since these studies did not report results regarding brain responses to neutral faces, they could not be included in a more comprehensive qualitative analysis such as a systematic review. We thus exclusively relied on the rigor of a more quantitative meta-analytical approach, which further ensures a principled weighing of individual studies.

The quality of included studies was assessed (by AMF) using a modified version of the quality assessment checklist employed in previous neuroimaging meta-analysis^25^ (see Table S1).

It should be noted that one of the included studies had a partial coverage of the brain^26^. There is a common consensus regarding the need to exclude studies employing region-of-interest analyses, to avoid biasing whole-brain results in favor of these regions. However, the brain coverage of this study was much wider than classical ROIs and encompassed all the brain regions typically involved in face processing.

Details regarding the task design, demographic variables, and included contrasts are reported in Table 1. The main objective, main results, and quality score of the included studies are reported in Table S2.

**Table 1:** Included studies investigating brain activation in response to neutral faces.

Reference	Kinship	Number of participants	Age mean (SD)	Male sex; No (%)	Field strength	Task used	Contrast included	T-maps/Coordinates
Diwadkar et al.^42^	First-degree relatives (offspring)	REL (17); HC (25)^a^	REL: 14.3 (3.1); HC: 14.6 (2.6)	REL: 12 (63); HC: 16 (67)	4 T	Emotion recognition (n-back)	Neutral > Control Condition	T-maps
Oertel et al. ^43^	First-degree relatives	REL (23); HC (27)	REL: 43.6 (14.3); HC: 34.2 (11.4)	REL: 5 (22); HC: 13 (48)	3 T	Associative memory	Neutral > Interstimulus Interval	Coordinates
Park et al.^44^	First-degree relatives (siblings or offspring)	REL (20); HC (17)	REL: 23.9 (5.6); HC: 23.06 (3.9)	REL: 7 (35); HC: 8 (47)	3 T	Gender recognition	Neutral > Interstimulus Interval	T-maps
Pirnia et al.^45^	First-degree relatives	REL (14); HC (30)	REL: 39.6 (11.8); HC: 29.3 (9.0)	REL: 5 (36); HC: 24 (80)	3 T	Associative memory	Neutral > Interstimulus Interval	Coordinates
Quarto et al.^34^	First-degree relatives (siblings)	REL (36); HC (56)	REL: 35.4 (10.1); HC: 31.4 (10.4)	REL: 13 (36); HC: 30 (54)	3 T	Gender recognition	Neutral > Interstimulus Interval^b^	T-maps
Spilka et al. ^35^	First-degree relatives (parents, siblings, offspring)	REL (27); HC (27)	REL: 41.2 (15.5); HC: 40.7 (11.1)	REL: 10 (37); HC: 13 (48)	3 T	Passive viewing	Neutral > Control Condition	T-maps
Wolf et al.^26^	First-degree relatives (parents and siblings)	REL (20); HC (25)	REL: 42.3 (14.8); HC: 39.0 (10.7)	REL: 9 (45); HC: 12 (48)	3 T	Emotion recognition	Neutral > Interstimulus Interval^b^	T-maps

*SD* standard deviation, *HC* healthy controls, *REL* healthy first-degree relatives, *T* Tesla.

^a^This number corresponds to the dataset sent by the authors which is slightly different from the dataset reported in the paper. This is because the authors retrieved the latest available dataset.

^b^The included contrast was not the one originally reported in the article but a newly generated one.

### Analyses

Using statistically unthresholded T-maps provided by the authors, we performed an image-based meta-analysis, which is more sensitive compared with more common coordinate-based meta-analyses^27,28^. This method accounts for effect sizes and is also sensitive to moderate subthreshold effects that are consistent across studies^29^. Such an approach is particularly critical in healthy first-degree relatives who are expected to exhibit more subtle brain differences compared to those found in patients with schizophrenia.

Some of the included contrasts were not reported in the original articles but were generated by the authors upon our request (see Table 1). This was necessary since several of the eligible studies included neutral faces only as a comparator for other conditions, without reporting brain responses to these stimuli in the original article.

#### Whole-brain analyses

Meta-analyses were performed using Seed-based d-mapping software based on permutation of subject images (SDM-PSI^30^, version 6.21). Traditional meta-analysis methods (e.g., Activation Likelihood Estimation, ALE, and Multilevel Kernel Density Analysis, MKDA) test for spatial convergence, evaluating in each voxel whether studies found activation more often in one group compared with another. In contrast, the SDM-PSI algorithm is regarded as more precise, as it tests for significant differences in activation between groups in each voxel^30^. Rather than convergence, this approach considers the consistency of activation or deactivation patterns across studies, providing a more robust assessment of common neural substrates. Even with a limited number of studies, consistent effects are more likely to be detected. Furthermore, it is possible to employ the SDM-PSI algorithm to conduct meta-analyses using whole-brain unthresholded T-maps, yielding a substantial gain in statistical power and sensitivity compared with coordinate-based meta-analyses^27,28^. Accordingly, the validation process of the SDM-PSI algorithm has indicated that a 100% sensitivity in detecting the proportion of activated voxels can be achieved by using the T-maps from at least three studies^30^.

Furthermore, the SDM-PSI approach offers several advantages, including a more straightforward interpretation of results, independence of voxel-level significance from the distribution of activation across the brain, and the ability to account for both activations and deactivations. Finally, this method employs a threshold-free cluster enhancement (TFCE) approach to address the multiple comparison issue, which compared with other thresholding techniques has the benefit of not defining an arbitrary cluster-forming threshold^31^.

We included a single contrast per study depicting a comparison between the perception of neutral faces versus a control condition or interstimulus interval (e.g., fixation cross, checkerboard pattern). We initially examined these contrasts separately in first-degree relatives and healthy controls (within-group meta-analysis). Then, the same contrasts were examined in first-degree relatives versus healthy controls (between-group meta-analysis). A total of 120 first-degree relatives and 150 healthy controls from five studies were included in these analyses.

Results were corrected for multiple comparisons across the whole brain using a TFCE-corrected threshold of pTFCE < 0.05.

#### Sensitivity and heterogeneity analyses

To assess the robustness of our findings, we conducted sensitivity analyses using a combined image- and coordinate-based meta-analysis. Specifically, through multiple imputations the SDM algorithm reconstructs estimated 3D statistical maps from coordinates^27,30^ (see Supplementary Methods for a description of this procedure). This process allows for the combination of statistical parametric maps, significantly enhancing sensitivity but less often available, with peak coordinates, more commonly reported in original articles.

In addition to the five studies that were included in our original analysis, we could include two additional studies that reported peak coordinates in their original articles (*n* = 7 studies), cumulating 157 first-degree relatives and 207 healthy controls.

Furthermore, in order to verify that our results were not partially driven by the inclusion of one study with partial brain coverage^26^, we ran an additional analysis excluding this study (*n* = 4 studies).

Additionally, to account for the potential variance that could be attributed to age and study quality, we conducted a supplementary analysis, including mean age and total quality score (as assessed by the quality assessment checklist, see Table S1; total quality score is reported in Table S2) as covariates in our model.

Finally, we examined the whole-brain heterogeneity map generated by SDM-PSI. This map contains a *I*² statistic for each voxel, which represents the percentage of total variation in effect size due to between-study heterogeneity rather than sampling error.

## Results

Whole-brain maps of within- and between-group meta-analyses as well as whole-brain maps of sensitivity analyses are available on NeuroVault [https://neurovault.org/collections/13917/].

### Whole-brain within-group meta-analysis

**Fig. 2:**
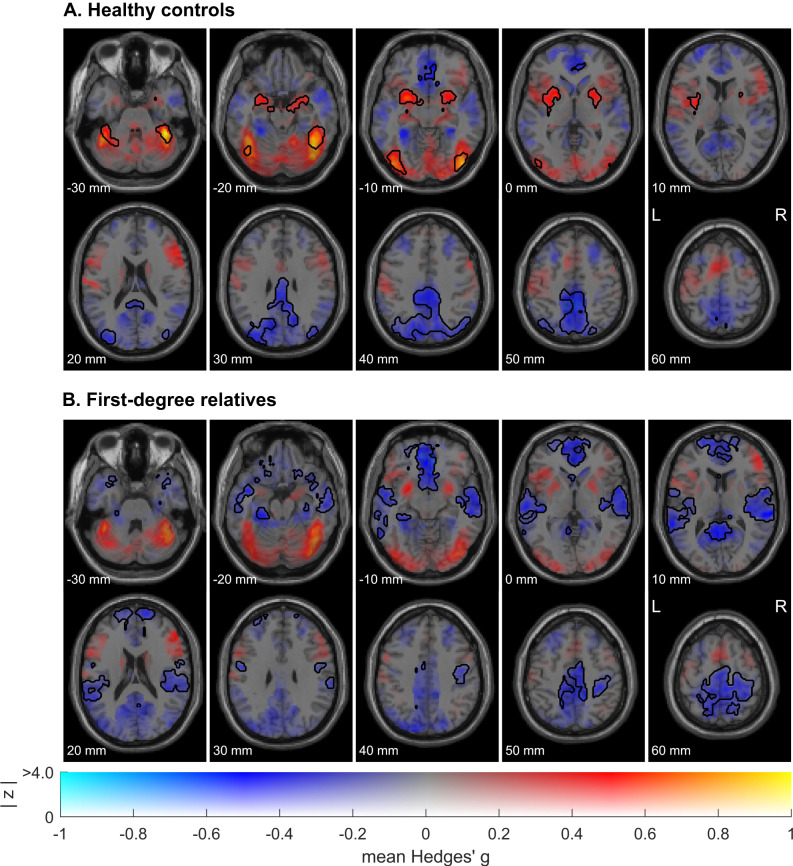
Image-based within-group meta-analysis. Combining functional neuroimaging studies investigating brain responses to neutral faces in healthy controls (*n* = 5 studies; 150 healthy controls) (**A**) and healthy first-degree relatives of patients with schizophrenia (*n* = 5 studies; 120 first-degree relatives) (**B**). These are dual-coded images^46,47^ in which color represents mean Hedges’ g (brain regions showing activations are depicted in red while deactivations are depicted in blue), and transparency represents *z*-values. Black line contours denote significant (de-)activations at a threshold of *p*_TFCE_ < 0.05.

In healthy controls, the meta-analysis of within-group T-maps revealed significant activations in several brain regions of the limbic system, such as bilateral amygdala and insula, as well as bilateral putamen and fusiform face area. Deactivations were observed in regions of the default mode network, such as bilateral posterior cingulate gyrus, superior parietal gyrus, occipital lobe, and pre-subgenual frontal cortex (Fig. 2A, see Table S3 for MNI coordinates).

In healthy first-degree relatives, no statistically significant brain activations were observed. However, it is worth noting that activations in the fusiform face area and amygdala were detected, although they did not reach the threshold for statistical significance. In contrast, relatives showed numerous significant deactivations, in the bilateral superior temporal gyrus, middle inferior temporal gyrus, precentral and postcentral gyrus, subgenual frontal cortex, and parahippocampal gyrus (Fig. 2B, see Table S3 for MNI coordinates).

### Whole brain between-group meta-analysis

**Fig. 3:**
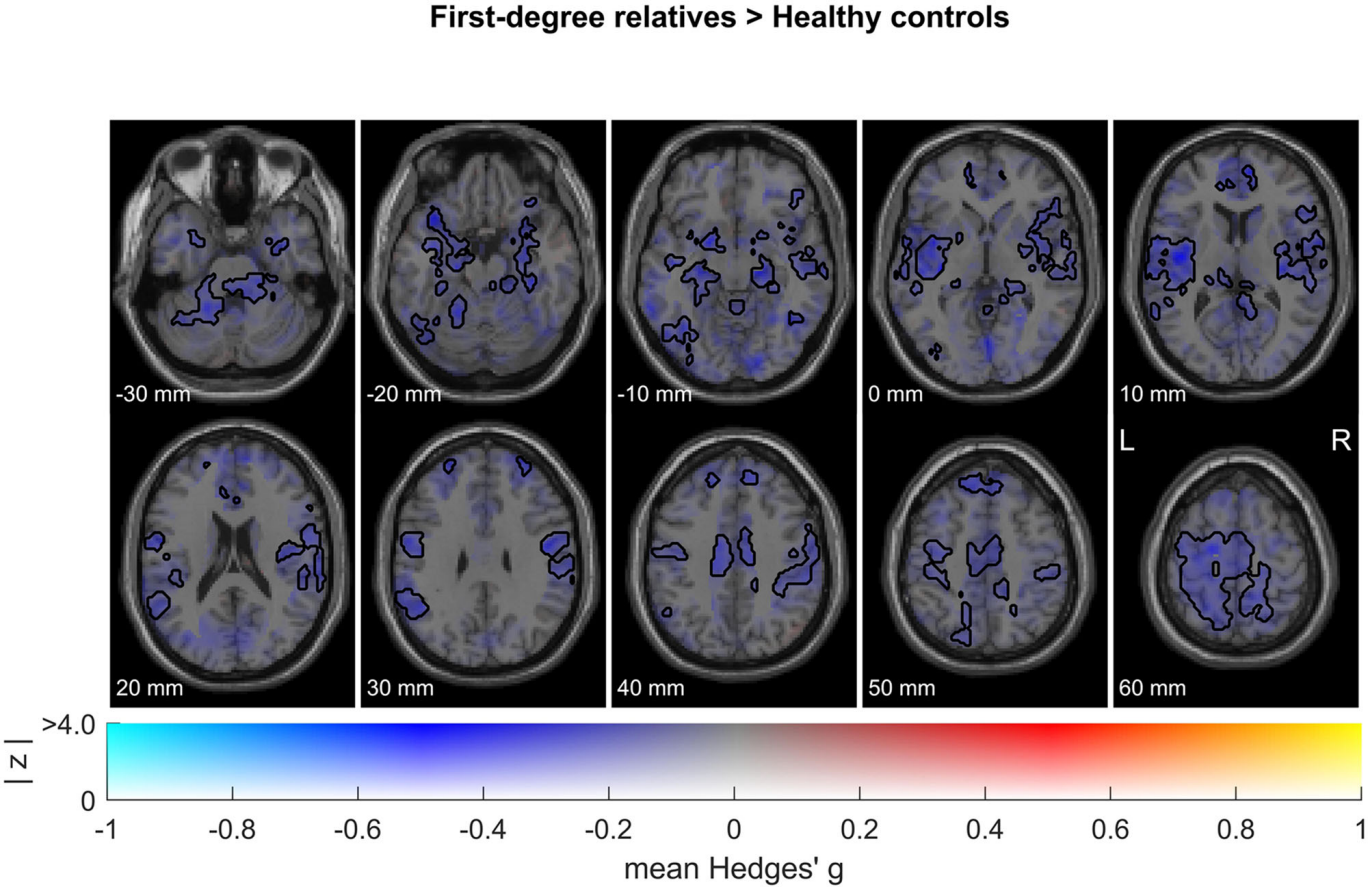
Image-based between-group meta-analysis. Combining functional neuroimaging studies investigating brain responses to neutral faces in healthy first-degree relatives of patients with schizophrenia versus healthy controls (*n* = 5 studies; 120 first-degree relatives and 150 healthy controls). These are dual-coded images^46,47^ in which color represents mean Hedges’ g (brain regions showing activations are depicted in red while deactivations are depicted in blue), and transparency represents *z*-values. Black line contours denote significant (de-) activations at a threshold of *p*_TFCE_ < 0.05.

First-degree relatives showed significantly decreased activations compared with healthy controls during the processing of neutral faces, particularly in limbic areas including the bilateral amygdala, hippocampus, and insula (Fig. 3, see Table S4 for MNI coordinates). Remarkably, among these regions, the amygdala was the only one showing a clear bilateral hypoactivation pattern, i.e., was significantly activated in healthy controls while not significantly activated in first-degree relatives. No increased activations were observed.

In forest plots, we have depicted the effect sizes of individual studies included in the present meta-analysis both for the left and right amygdala, which were defined independently as anatomical regions of interest using the Melbourne Subcortex Atlas^32^. The pattern of results is very similar in the left and right amygdala, reducing the chances of a false positive result (see Fig. S1). Furthermore, publication bias was assessed in the left and right amygdala, first through visual inspection of the funnel plot, which represents the precision of each study as a function of its effect size. In the absence of publication bias, studies are expected to be symmetrically distributed (see Fig. S2 for a visual inspection of publication bias). Secondly, we used Egger’s regression test, a quantitative method that tests for the presence of asymmetry in the funnel plot, which was not significant for either the left (*t* = −0.28, *p* = 0.7996) or right (*t* = −1.32, *p* = 0.277) amygdala, indicating reasonable symmetry of the funnel plot and thus no evidence of a publication bias.

### Sensitivity and heterogeneity analyses

The results of the combined image- and coordinate-based meta-analysis largely replicated the findings of the main analysis (see Fig. S3).

Moreover, when excluding the study with partial brain coverage as well as when running the main analysis by adding mean age and study quality as covariates, the main results remained similar (see Figs. S4 and  S5).

Despite low heterogeneity observed across most of the brain as indicated by the heterogeneity map, we identified some clusters exhibiting substantial heterogeneity (see Fig. S6). However, these clusters were mostly non-overlapping with the peak of (de-)activations observed in the between-group comparison.

## Discussion

In this image-based meta-analysis, we compared brain responses to neutral faces between healthy first-degree relatives of patients with schizophrenia and healthy controls.

It is now well-established that neutral face processing in physiological conditions involves not only activation of the posterior network (which includes the visual and fusiform face areas), but also of other core structures such as the amygdala^33^. The within-group meta-analysis showed robust activations in these regions in healthy controls, whereas in first-degree relatives these activations did not reach statistical significance, suggesting that these areas are activated to a lesser extent. This result was confirmed in the between-group meta-analysis, which revealed significant hypoactivation of the amygdala and limbic regions in first-degree relatives compared with healthy controls. Notably, included studies did not report a difference in performance on the behavioral task between first-degree relatives and healthy controls (besides one study that found decreased accuracy specifically for identification of sad faces^26^). This finding challenges the possibility that the observed differences could be attributed to a lack of engagement or involvement in the task by first-degree relatives.

Our findings are particularly noteworthy in light of previous studies in patients with schizophrenia, indicating both misattribution of emotional valence to neutral faces^3–5^ and emotional brain hypersensitivity to emotionally neutral stimuli^7^ (although this result in patients could not be replicated in the present meta-analysis, as only 2 out of 5 studies included both first-degree relatives and patients with schizophrenia^34,35^). Given prevailing work reporting no abnormalities in recognition of neutral faces in healthy first-degree relatives^13–16^ and given the crucial role of the amygdala and limbic regions in the attribution of emotional and motivational significance, the observed decrease of activation in these regions in healthy first-degree relatives may represent a physiological protective marker against the onset of schizophrenia disorder. Indeed, it should be noted that only a minority of first-degree relatives undergo the development of schizophrenia (i.e., transition rate of 13% at 1-year follow-up^36^). Furthermore, the majority of individual studies included in this meta-analysis have focused on first-degree relatives who passed the median age of onset for schizophrenia (i.e., 25 years^37^) and are thus less susceptible to developing schizophrenia. Accordingly, while healthy first-degree relatives share about half of their genes with their affected sibling, other genetic factors, possibly related to their remaining genetic heritage, may have protective potential against polygenic schizophrenia-associated variants. The search for risk markers of schizophrenia has so far largely overlooked the search for protective factors^38^. Yet, both have the potential to help clinicians better predict the transition to psychosis and inform therapeutic targets for prevention. Indeed, recent research has shown a growing interest in these genetic factors, leading to a shift in focus from a polygenic risk score to what is defined as a “polygenic resilience score” for schizophrenia^39^.

Consistent with our findings, recent research has outlined a cerebral circuit underpinning protective factors against the established adversities of mental illnesses such as schizophrenia, within which the capacity to downregulate the amygdala constitutes a key mechanism^40^. In line with his idea, recent studies employing real-time fMRI neurofeedback of the amygdala have demonstrated that training the downregulation of amygdala activity facilitates emotion regulation^41^. This ability to regulate emotions aids in coping with emotionally challenging situations and, consequently, mitigating stress -an influential factor in the onset of schizophrenia. Interestingly, previous studies investigating brain connectivity in response to neutral face processing in offspring of patients with schizophrenia have reported increased cortico-limbic top-down modulation^42^.

The correlation suggested between reduced engagement of the limbic system and reduced vulnerability to schizophrenia warrants further exploration. Particularly, future longitudinal studies are imperative to investigate whether decreased activation of limbic regions is specific to individuals at familial risk who do not transition to schizophrenia. This exploration is pivotal in discerning the genuine protective role of this trait, which might hold promise as a potential predictive factor for prognosis. A critical initial step towards elucidating this point involves clarifying the relationship between limbic responses and schizophrenia symptomatology. Prior studies have suggested that heightened limbic activation in individuals with schizophrenia may contribute to positive symptoms, particularly paranoid or delusional ideation, but failed to provide empirical evidence supporting this relationship^7^. Elucidating this link may help in discerning whether the present finding of diminished limbic activation in first-degree relatives represents a protective factor, potentially explaining an absence of symptomatology in these relatives.

These results should be considered in light of several limitations. First, we could only include a limited number of studies (*n* = 7, among which 5 unthresholded T-maps). This is mainly due to the limited availability of fMRI studies exploring neutral processing in first-degree relatives. Indeed, merely 5 studies have reported such an analysis in their original publication (see Table 1). Yet, none of these studies primarily focused on examining brain activity related to neutral stimuli (see Table S2 for an overview of the main objective and results of the included studies). In this context, our approach consisted of contacting the corresponding authors of all the studies that employ neutral faces in their task design, regardless of whether activations to neutral faces were reported in the original article. This procedure allowed us to collect results from 2 additional studies. Furthermore, although the number of included studies was limited, the use of unthresholded T-maps significantly increases accuracy, as compared with meta-analysis relying on peak coordinates. Finally, it has been shown that using SDM-PSI, a 100% sensitivity in detecting the proportion of activated voxels can be reached by using at least the T-maps from 3 studies^30^. Second, it is important to emphasize that schizophrenia is a highly heterogeneous disorder and that this heterogeneity poses a substantial challenge to our understanding. This variability is not only evident among patients but is equally pronounced among healthy relatives. Our meta-analysis, while providing valuable insights, must be interpreted in light of this inherent heterogeneity. It also underscores the need for future studies to delve deeper into the intricacies of clinical variations within both patient and familial populations. Third, one of the included studies has a partial coverage of the brain^26^. However, we largely replicated the results of the main analysis while excluding this study (see Fig. S4). Moreover, since SDM-PSI software does not test for spatial convergence, but rather for significant differences in activation, the inclusion of studies with partial brain coverage does not lead to an increase of false positive results.

## Conclusion

Despite these limitations, this study constitutes the first attempt to meta-analytically investigate the brain processing of neutral faces in healthy first-degree relatives of patients with schizophrenia. Compared with healthy controls, we have reported widespread decreased activations in first-degree relatives, particularly in regions of the limbic system. This pattern of decreased activations contrasts with the one observed in patients with schizophrenia, showing increased activations of limbic regions^7^, and possibly suggesting the presence of protective factors. The findings of this meta-analysis warrant and encourage future investigations into the brain responses to neutral stimuli among first-degree relatives, a facet of research that has been significantly overlooked. Furthermore, our results align with those of Dugré and colleagues^7^ in cautioning against the use of neutral faces as a baseline control condition in future fMRI studies and extend this vigilance to populations of first-degree relatives. Indeed, neutral faces may elicit different brain responses in these various populations of subjects and may confound results.

### Supplementary information

Supplementary Material

## Data Availability

Whole-brain maps of within- and between-group meta-analyses as well as whole-brain maps of sensitivity analyses are available on NeuroVault [https://neurovault.org/collections/13917/].
