# Supplementary material for: Blunted brain responses to neutral faces in healthy first-degree relatives of patients with schizophrenia: an image-based fMRI meta-analysis
Source: Schizophrenia (Heidelb). 2024 Mar 19;10(1):38. doi: 10.1038/s41537-024-00452-6 (PMC10951276; doi:10.1038/s41537-024-00452-6)
Supplement: Supplementary file 1 — Supplementary Material [file 41537_2024_452_MOESM1_ESM.pdf]

SUPPLEMENTARY MATERIAL

Blunted brain responses to neutral faces in healthy first-degree relatives of patients with schizophrenia: an image-based fMRI meta-analysis

Fiorito *et al.*

Contents:

Supplementary Methods 2
Literature search 2
Study exclusion 2
Sensitivity analysis combining image- and coordinate-based meta-analysis 2
Table S1. Quality assessment checklist 3
Table S2. Main objective and main results of the included studies 4
Table S3. MNI coordinates of within-group meta-analysis 5
Table S4. MNI coordinates of between-group meta-analysis 6
Supplementary Figure S1. Forest plots 7
Supplementary Figure S2. Funnel plots 8
Supplementary Figure S3. Combined image- and coordinate-based between-group meta-analysis 9
Supplementary Figure S4. Meta-analysis without studies with partial brain coverage 10
Supplementary Figure S5. Meta-analysis (with mean age and quality of the study as covariates) 11
Supplementary Figure S6. Whole-brain heterogeneity map 12
References 13

Supplementary Methods

Literature search

PubMed search employed a combination of the following keywords: ("schizophren*" OR "psychosis") AND ("relatives" OR "first-degree" OR "siblings" OR "twins" OR "brothers" OR "sisters" OR "offspring" OR "parents" OR "genetic risk") AND ("neuroimaging" OR "fMRI" OR "functional Magnetic Resonance Imaging") AND ("emotion*" OR "affect" OR "mood" OR "face" OR "facial").

Web of Science review was based on the following keywords: TS=(schizophren* OR psychosis) AND TS=(relatives OR first-degree OR siblings OR twins OR brothers OR sisters OR offspring OR parents OR genetic risk) AND TS=(neuroimaging OR fMRI OR functional magnetic resonance) AND TS=(emotion* OR affect* OR mood OR face OR facial).

Study exclusion

50 studies were excluded due to the following reasons:

- 12 studies did not include neutral faces[1–12]
- 11 studies used non-relevant task design (1 structural magnetic resonance imaging[13], 2 non-visual stimuli[14,15], 2 resting state[16,17], 6 studies employed scenes or stories instead of faces[18–23])
- 7 were conference abstracts[24–30]
- 7 studies employed the same dataset as in another included study[31–37]
- 6 studies were systematic reviews or meta-analyses[38–43]
- 6 study did not include a group of first-degree relatives[44–49]
- 1 study was a method paper[50]

Sensitivity analysis combining image- and coordinate-based meta-analysis

In order to assess the robustness of our image-based meta-analytic results, we performed a sensitivity analysis wherein we added 2 studies reporting brain coordinates. The SDM methodology allows to conduct such meta-analyses that combines 3D statistical images and peak coordinates by leveraging its ability to reconstruct 3D statistical maps from image coordinates. This involves converting t-values of peak coordinates into effect sizes and gradually imputing effect sizes for surrounding voxels, considering proximity to peaks and accounting for estimation inaccuracies through multiple imputations[51,52].

Table S1. Quality assessment checklist adapted from Zheng et al., 2018[53]. For each study and each item, a score of 0/0.5/1 is assigned (0 if criteria not met, 0.5 if partially met, 1 if clearly met). Total score is 12 out of 12.

**Category 1: Sample characteristics**
Patients were evaluated with specific standardized diagnostic criteria
Important demographic data (age, gender, and education) were reported with mean (or median) and standard deviations (or range))
Healthy comparison subjects were evaluated to exclude psychiatric and medical illnesses
Important clinical variables were reported with mean (or median) and standard deviations (or range))
Sample size per group > 10
**Category 2: Methodology and reporting**
Whole brain analysis was automated with no a-priori regional selection
Magnet strength at least 1.5T
Whole brain coverage of fMRI scans
The acquisition and preprocessing techniques were clearly described so that they could be reproduced
Coordinates reported in a standard space
Significant results are reported after correction for multiple testing using a standard statistical procedure (FDR, FWE or permutation-based methods)
Conclusions were consistent with the results obtained and the limitations were discussed

Table S2. Main objective and main results of the included studies

Reference	Main objective	Main results	Quality score
Diwadkar et al., 2012[54]	To investigate effective brain connectivity associated with emotional processing in schizophrenia	Abnormal connectivity during emotion processing in offspring of patients with schizophrenia	11
Oertel et al., 2019[55]	To investigate associative memory in schizophrenia	Abnormal brain activity during retrieval in patients with schizophrenia and to a lesser degree in first-degree relatives	12
Park et al., 2016[56]	To investigate implicit emotion processing in schizophrenia	Abnormal brain activity to fearful and neutral faces in first-degree relatives	11
Pirnia et al., 2015[57]	To investigate associative memory in schizophrenia	Abnormal brain activity to successful encoding in patients with schizophrenia	11.5
Quarto et al., 2018[58]	To investigate effective brain connectivity associated with emotional processing in schizophrenia	Abnormal connectivity during emotion processing in both schizophrenia patients and first-degree relatives	11.5
Spilka et al., 2015[59]	To investigate implicit emotion processing in schizophrenia	Abnormal brain activity to emotions in first-degree relatives and patients with schizophrenia.	11.5
Wolf et al., 2011[60]	To identify and modulate emotion processing in schizophrenia	Abnormal brain activity to emotion identification induced by GABAergic modulation in first-degree relatives	11

Table S3. MNI coordinates of within-group meta-analysis. We employed the Hammersmith brain atlas (n30r83, © Copyright Imperial College of Science, Technology and Medicine 2007. All rights reserved[61]) in order to determine the name of brain structures.

Brain region	Hemisphere	MNI (x,y,z)	SDM-Z
Healthy controls *- Activations*			
Amygdala	Left	-22, -3, -16	4.5863
Amygdala	Right	22, -3, -14	4.4872
Insula	Left	-32, 4, -10	5.2278
Insula	Right	32, 4, -4	4.8726
Putamen	Left	-22, 8, 6	5.4896
Putamen	Right	26, 7, -2	5.7281
Occipital lobe (Fusiform Face Area)	Left	-44, -81, -5	4.0052
Occipital lobe (Fusiform Face Area)	Right	42, -86, -8	3.93463
Healthy controls *- Deactivations*			
Posterior cingulate gyrus	Left	-4, -31, 26	3.9956
Posterior cingulate gyrus	Right	6, -33, 28	3.9061
Superior parietal gyrus	Left	-12, -61, 44	3.3147
Superior parietal gyrus	Right	12, -70, 40	3.6446
Occipital lobe	Left	-30, -82, 24	3.7414
Occipital lobe	Right	40, -84, 24	3.3893
Pre-subgenual frontal cortex	Left	-10, 26, -12	4.4617
Pre-subgenual frontal cortex	Right	8, 28, -10	4.6346
First-degree relatives *- Deactivations*			
Superior temporal gyrus	Left	-64, -23, 4	3.7838
Superior temporal gyrus	Right	59, -28, 8	5.0942
Middle inferior temporal gyrus	Left	-54, -12, -16	2.5841
Middle inferior temporal gyrus	Right	54, -20, -16	4.0745
Precentral gyrus	Left	-11, -32, 66	3.3799
Precentral gyrus	Right	2, -28, 66	3.6165
Postcentral gyrus	Left	-14, -32, 68	3.5393
Postcentral gyrus	Right	34, -32, 52	3.6572
Subgenual frontal cortex	Left	1, 22, -12	3.5019
Subgenual frontal cortex	Right	-12, 22, -14	3.4752
Parahippocampal gyrus	Left	-22, -30, -22	4.4278
Parahippocampal gyrus	Right	34, -21, -22	3.3432

Table S4. MNI coordinates of between-group meta-analysis. We employed the Hammersmith brain atlas (n30r83, © Copyright Imperial College of Science, Technology and Medicine 2007. All rights reserved[61]) in order to determine the name of brain structures.

Brain region	Hemisphere	MNI (x,y,z)	SDM-Z
First-degree relatives < Healthy controls			
Hippocampus	Left	-26, -22, -12	-2.8437
Parahippocampal gyrus	Left	-20, -18, -20	-2.8497
Hippocampus	Right	28, -12, -16	-3.141
Insula	Left	-40, -10, 8	-3.96045
Insula	Right	64, 20, -4	-2,423
Putamen	Left	-17, 4, -9	-2.60312
Amygdala	Right	28, 0, -26	-2.8438
Amygdala	Left	-22, -4, -24	-2.18346
Inferiolateral parietal lobe	Left	-46, -52, 28	-2.6742
Inferiolateral parietal lobe	Right	52, -27, 39	-2.10457
Superior parietal gyrus	Right	8, -52, 68	-3.47912
Superior parietal gyrus	Left	-22, -52, 62	-2.34547
Cerebellum	Left	-18, -48, -32	-2.85079
Cerebellum	Right	16, -36, -34	-2.2929
Inferior frontal gyrus	Right	52, 22, 12	-2.36139
Precentral gyrus	Right	2, -24, 68	-3.25278
Precentral gyrus	Left	-16, -24, 74	-2.70431
Postcentral gyrus	Left	-10, -30, 76	-2.79808
Postcentral gyrus	Right	8, -31, 76	-2.76954
Posterior cingulate gyrus	Left	-10, -22, 40	-2.41395
Posterior cingulate gyrus	Right	10, -7, 42	-2.37083

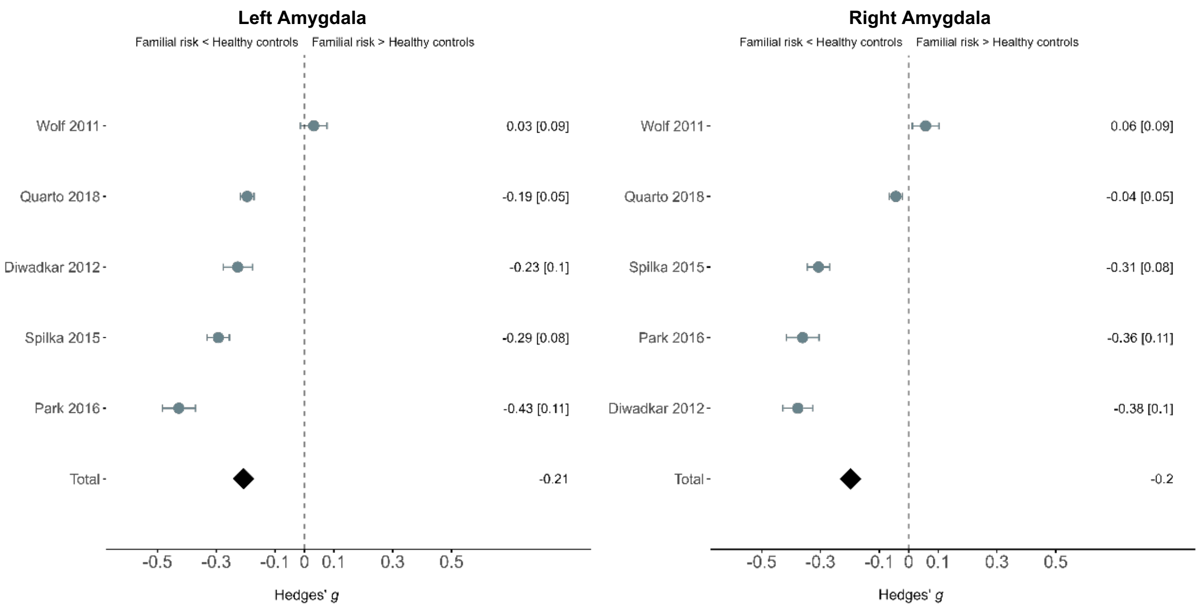

Supplementary Figure S1. Forest plots depicting the mean ± variance of effect sizes for group comparison in the left and right amygdala (defined using the Melbourne Subcortex Atlas[62]). The black diamonds represent the overall effect size.

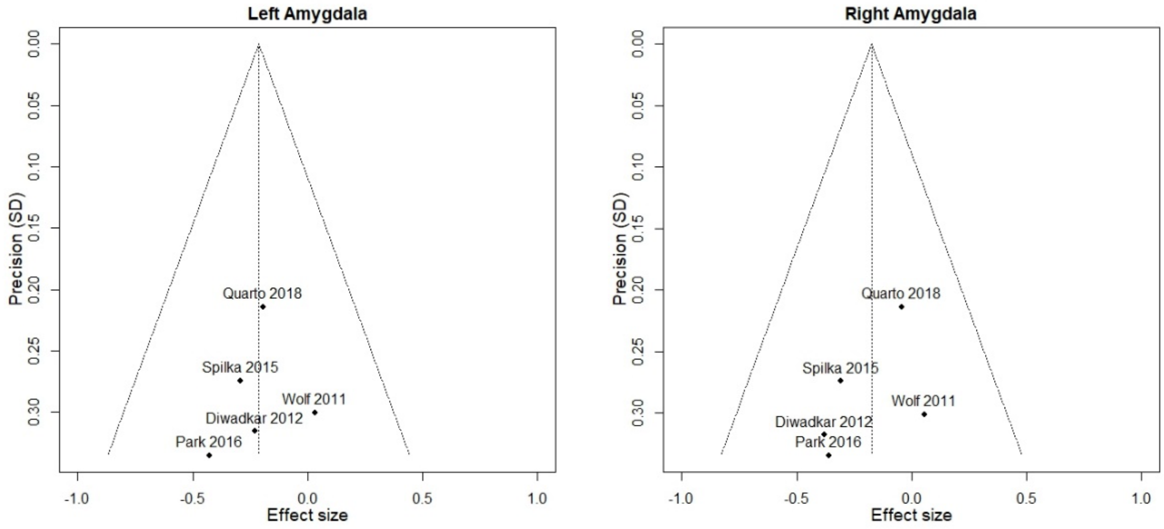

Supplementary Figure S2. Funnel plots in left and right amygdala. The symmetrical distribution of studies suggests no evidence for publication bias.

First-degree relatives > Healthy controls

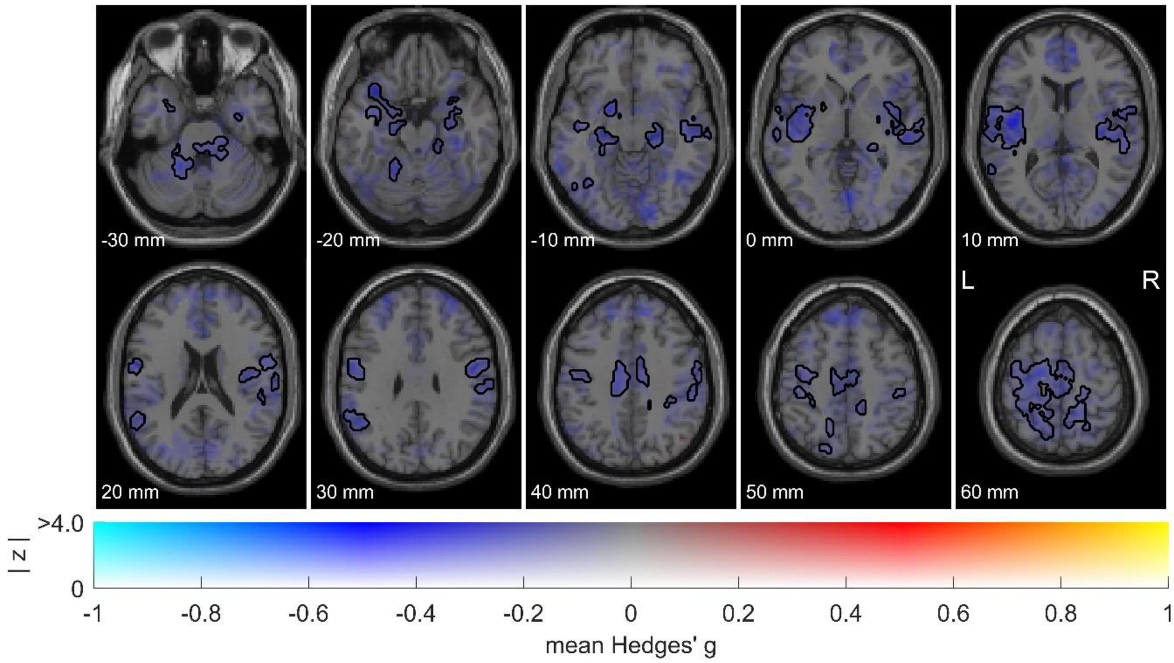

Supplementary Figure S3. Combined image- and coordinate-based between-group meta-analysis of functional neuroimaging studies investigating brain responses to neutral faces in healthy first-degree relatives of patients with schizophrenia versus healthy controls (n = 7 studies; 157 first-degree relatives and 207 healthy controls). *These are dual-coded* images[63,64] *in which color represents mean Hedges' g (brain regions showing activations are depicted in red while deactivations are depicted in blue), and transparency represents z-values. Black line contours denote significant (de-)activations at* $p_{TFCE} < 0.05$.

First-degree relatives > Healthy controls

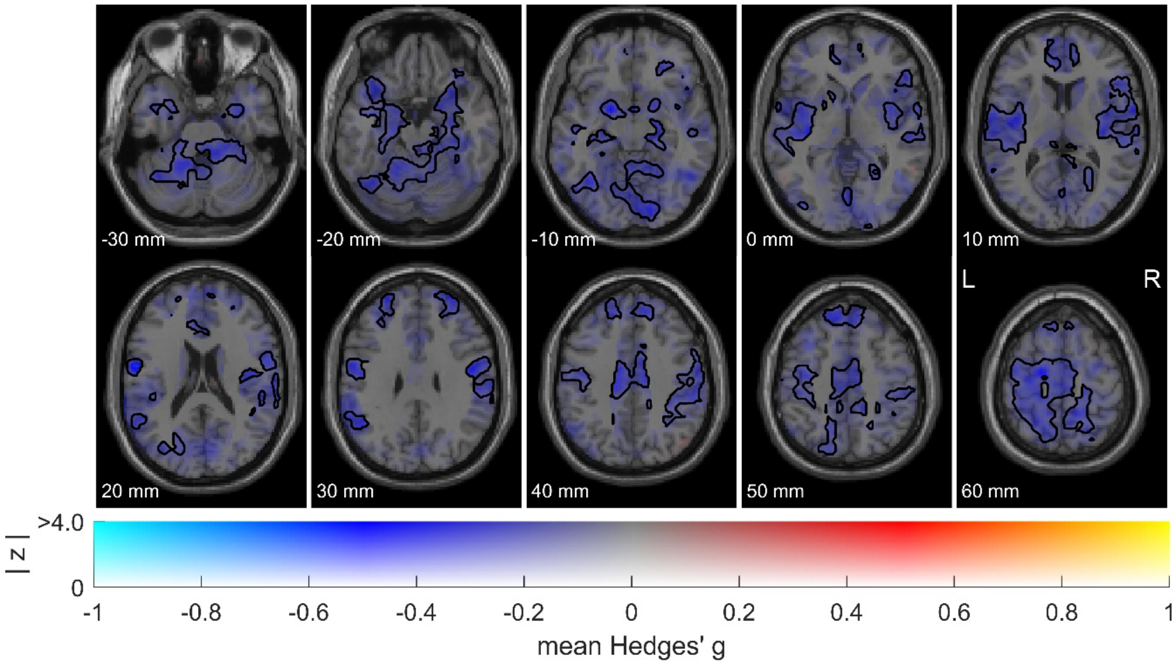

Supplementary Figure S4. Meta-analysis without studies with partial brain coverage (1 study excluded[60], n = 4 studies; 100 first-degree relatives and 125 healthy controls). *These are dual-coded* images[63,64] *in which color represents mean Hedges' g (brain regions showing activations are depicted in red while deactivations are depicted in blue), and transparency represents z-values. Black line contours denote significant (de-)activations at* $p_{TFCE} < 0.05$.

First-degree relatives > Healthy controls

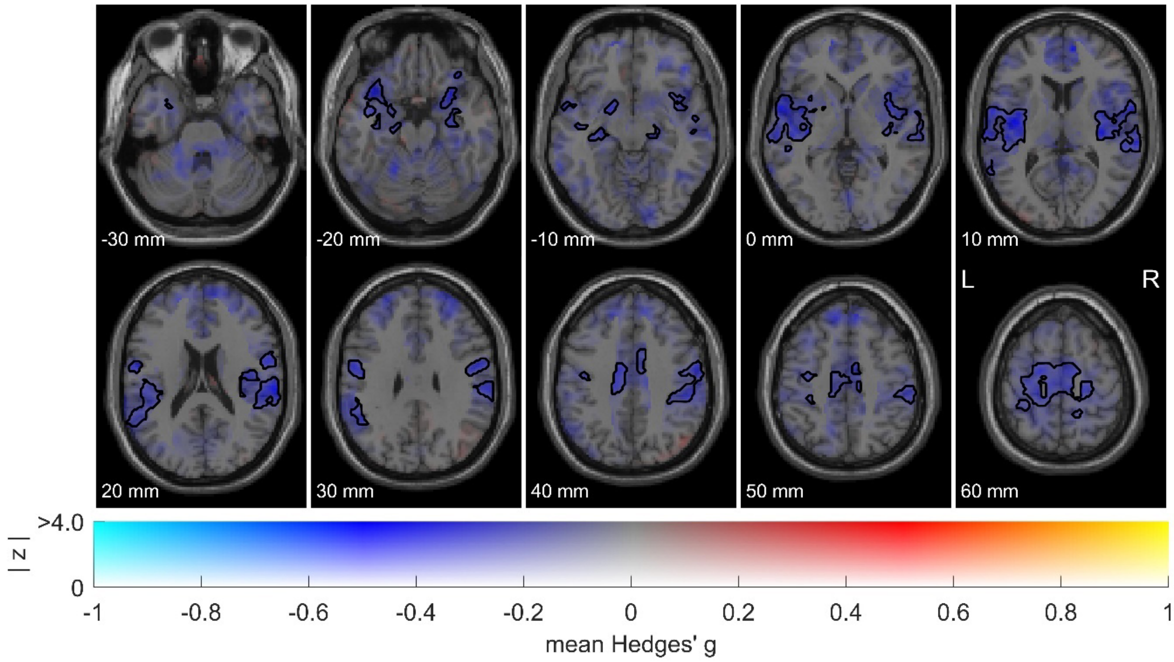

Supplementary Figure S5. Meta-analysis (with mean age and quality of the study as covariates) of functional neuroimaging studies investigating brain responses to neutral faces in healthy first-degree relatives of patients with schizophrenia versus healthy controls (n = 5 studies; 120 first-degree relatives and 150 healthy controls). *These are dual-coded* images[63,64] *in which color represents mean Hedges' g (brain regions showing activations are depicted in red while deactivations are depicted in blue), and transparency represents z-values. Black line contours denote significant (de-)activations at* $p_{TFCE} < 0.05$.

First-degree relatives > Healthy controls

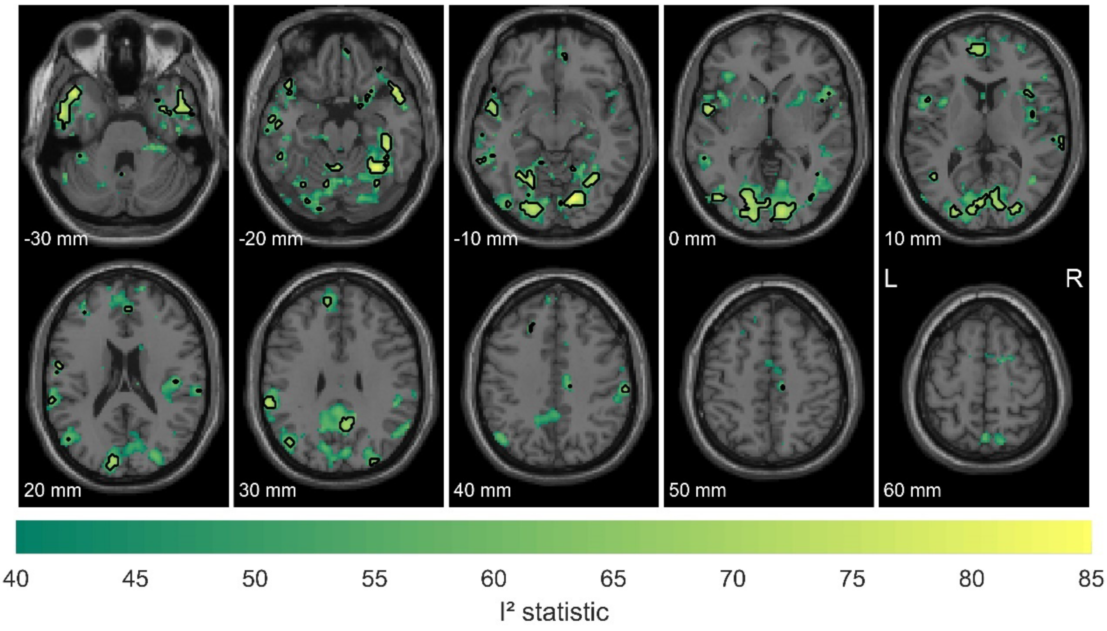

Supplementary Figure S6. Whole-brain heterogeneity map. Heterogeneity map displaying for each voxel the I^2 statistic. Since an I^2 statistic lower than 40% is commonly interpreted as low heterogeneity[65], the map was thresholded at $I^2 > 40\%$. Black line contours denote clusters showing at least substantial heterogeneity ($I^2 > 60\%$).

References

1. Cao H, Bertolino A, Walter H, et al. Altered functional subnetwork during emotional face processing: a potential intermediate phenotype for schizophrenia. *JAMA Psychiatry*. 2016;73(6):598-605.
2. de Achával D, Villarreal MF, Costanzo EY, et al. Decreased activity in right-hemisphere structures involved in social cognition in siblings discordant for schizophrenia. *Schizophr Res*. 2012;134(2-3):171-179. doi:10.1016/j.schres.2011.11.010
3. Dodell-Feder D, DeLisi LE, Hooker CI. Neural disruption to theory of mind predicts daily social functioning in individuals at familial high-risk for schizophrenia. *Soc Cogn Affect Neurosci*. 2014;9(12):1914-1925. doi:10.1093/scan/nst186
4. Falkenberg I, Chaddock C, Murray RM, et al. Failure to deactivate medial prefrontal cortex in people at high risk for psychosis. *Eur Psychiatry J Assoc Eur Psychiatr*. 2015;30(5):633-640. doi:10.1016/j.eurpsy.2015.03.003
5. Habel U, Klein M, Shah NJ, et al. Genetic load on amygdala hypofunction during sadness in nonaffected brothers of schizophrenia patients. *Am J Psychiatry*. 2004;161(10):1806-1813. doi:10.1176/ajp.161.10.1806
6. Mohnke S, Erk S, Schnell K, et al. Theory of mind network activity is altered in subjects with familial liability for schizophrenia. *Soc Cogn Affect Neurosci*. 2016;11(2):299-307. doi:10.1093/scan/nsv111
7. Pulkkinen J, Nikkinen J, Kiviniemi V, et al. Functional mapping of dynamic happy and fearful facial expressions in young adults with familial risk for psychosis - Oulu Brain and Mind Study. *Schizophr Res*. 2015;164(1-3):242-249. doi:10.1016/j.schres.2015.01.039
8. Rasetti R, Mattay VS, Wiedholz LM, et al. Evidence that altered amygdala activity in schizophrenia is related to clinical state and not genetic risk. *Am J Psychiatry*. 2009;166(2):216-225. doi:10.1176/appi.ajp.2008.08020261
9. Sambataro F, Mattay VS, Thurin K, et al. Altered Cerebral Response During Cognitive Control: A Potential Indicator of Genetic Liability for Schizophrenia. *Neuropsychopharmacology*. 2013;38(5):846-853. doi:10.1038/npp.2012.250
10. Schneider M, Walter H, Moessnang C, et al. Altered DLPFC–Hippocampus Connectivity During Working Memory: Independent Replication and Disorder Specificity of a Putative Genetic Risk Phenotype for Schizophrenia. *Schizophr Bull*. 2017;43(5):1114-1122. doi:10.1093/schbul/sbx001
11. Straub RE, Lipska BK, Egan MF, et al. Allelic variation in GAD1 (GAD67) is associated with schizophrenia and influences cortical function and gene expression. *Mol Psychiatry*. 2007;12(9):854-869.
12. Jimenez AM, Clayson PE, Hasratian AS, et al. Neuroimaging of social motivation during winning and losing: Associations with social anhedonia across the psychosis spectrum. *Neuropsychologia*. 2023;188:108621. doi:10.1016/j.neuropsychologia.2023.108621
13. Guimond S, Mothi SS, Makowski C, Chakravarty MM, Keshavan MS. Altered amygdala shape trajectories and emotion recognition in youth at familial high risk of schizophrenia who develop psychosis. *Transl Psychiatry*. 2022;12(1):1-8. doi:10.1038/s41398-022-01957-3
14. Nook EC, Dodell-Feder D, Germine LT, Hooley JM, DeLisi LE, Hooker CI. Weak dorsolateral prefrontal response to social criticism predicts worsened mood and symptoms following social conflict in people at familial risk for schizophrenia. *NeuroImage Clin*. 2018;18:40-50. doi:10.1016/j.nicl.2018.01.004
15. Schneider F, Habel U, Reske M, Toni I, Falkai P, Shah NJ. Neural substrates of

olfactory processing in schizophrenia patients and their healthy relatives. *Psychiatry Res*. 2007;155(2):103-112. doi:10.1016/j.pscychresns.2006.12.004
16. Fornito A, Harrison BJ, Goodby E, et al. Functional dysconnectivity of corticostriatal circuitry as a risk phenotype for psychosis. *JAMA Psychiatry*. 2013;70(11):1143-1151. doi:10.1001/jamapsychiatry.2013.1976
17. Tian L, Meng C, Yan H, et al. Convergent evidence from multimodal imaging reveals amygdala abnormalities in schizophrenic patients and their first-degree relatives. *PloS One*. 2011;6(12):e28794. doi:10.1371/journal.pone.0028794
18. Fahim C, Stip E, Mancini-Marıe A, Beauregard M. Genes and memory: the neuroanatomical correlates of emotional memory in monozygotic twin discordant for schizophrenia. *Brain Cogn*. 2004;55(2):250-253.
19. Hart SJ, Bizzell J, McMahon MA, Gu H, Perkins DO, Belger A. Altered fronto-limbic activity in children and adolescents with familial high risk for schizophrenia. *Psychiatry Res*. 2013;212(1):19-27. doi:10.1016/j.pscychresns.2012.12.003
20. Takano Y, Aoki Y, Yahata N, et al. Neural basis for inferring false beliefs and social emotions in others among individuals with schizophrenia and those at ultra-high risk for psychosis. *Psychiatry Res Neuroimaging*. 2017;259:34-41. doi:10.1016/j.pscychresns.2016.11.003
21. van der Meer L, Swart M, van der Velde J, et al. Neural correlates of emotion regulation in patients with schizophrenia and non-affected siblings. *PloS One*. 2014;9(6):e99667. doi:10.1371/journal.pone.0099667
22. van Buuren M, Vink M, Rapcencu AE, Kahn RS. Exaggerated brain activation during emotion processing in unaffected siblings of patients with schizophrenia. *Biol Psychiatry*. 2011;70(1):81-87. doi:10.1016/j.biopsych.2011.03.011
23. van Leeuwen JMC, Vink M, Fernández G, et al. At-risk individuals display altered brain activity following stress. *Neuropsychopharmacology*. 2018;43(9):1954-1960. doi:10.1038/s41386-018-0026-8
24. Barbour T. Increased Anhedonia in Adolescent Schizophrenia Offspring Predicts Hypo-Responsivity of Subgenual Frontal Cortex to Positive Stimuli. *Biol Psychiatry*. 2010;67(9):249S-249S.
25. Belger A, Andersen EH, Campbell AM, et al. Aberrant ERP Measures of Attention to Emotion Associated with Abnormal Temporo-Limbic FMRI Activation in Schizophrenia and First Degree Relatives: Society of Biological Psychiatry 69th Annual Scientific Meeting. In: ; 2014:338S-338S. doi:10.1016/j.biopsych.2014.03.016
26. Gonzalez-Garrido A, Quinones-Beltran S, Epelbaum JB, Gallardo-Moreno G, Gomez-Velazquez F. Working Memory Processing of Emotional Stimuli in Young Individuals with High Genetic Risk of Schizophrenia. *Psychophysiology*. 2019;56:S64-S64.
27. Hart SJ, Bizzell J, Woodlief J, Belger A. Neuroimaging executive-emotional processing in adolescents at genetic risk for schizophrenia. *Biol Psychiatry*. 2008;63(7):220S-220S.
28. Mirzakhanian H, Jahshan C, Light G, Nunag J, Roman PD, Cadenhead KS. Automatic Sensory Discrimination and Emotion Recognition in Prodromal and First-Episode Schizophrenia. *Biol Psychiatry*. 2011;69(9):234S-235S.
29. Pruitt P, Murphy E, Keshavan M, et al. Disordered Functional Maturation of the Amygdala During Adolescence: Fmri Studies of Affective Judgment in Schizophrenia Offspring. *Schizophr Bull*. 2009;35:171-172.
30. Pruitt PJ, Murphy ER, Keshavan MS, Rajan U, Zajac-Benitez C, Diwadkar VA. Adolescent Offspring of Schizophrenia Patients Show Reduced Amygdala-Related

Modulation of Memory Systems During Affective Memory Task. *Biol Psychiatry*. 2010;67(9):154S-154S.
31. Barbour T, Murphy E, Pruitt P, et al. Reduced intra-amygdala activity to positively valenced faces in adolescent schizophrenia offspring. *Schizophr Res*. 2010;123(2):126-136. doi:10.1016/j.schres.2010.07.023
32. Barbour T, Pruitt P, Diwadkar VA. fMRI responses to emotional faces in children and adolescents at genetic risk for psychiatric illness share some of the features of depression. *J Affect Disord*. 2012;136(3):276-285. doi:10.1016/j.jad.2011.11.036
33. Goghari VM, Sanford N, Spilka MJ, Woodward TS. Task-Related Functional Connectivity Analysis of Emotion Discrimination in a Family Study of Schizophrenia. *Schizophr Bull*. 2017;43(6):1348-1362. doi:10.1093/schbul/sbx004
34. Goldschmidt MG, Villarreal MF, de Achával D, et al. Cluster B personality symptoms in persons at genetic risk for schizophrenia are associated with social competence and activation of the right temporo-parietal junction during emotion processing. *Psychiatry Res*. 2014;221(1):30-36. doi:10.1016/j.pscychresns.2013.10.008
35. Mahadevan AS, Cornblath EJ, Lydon-Staley DM, et al. Alprazolam modulates persistence energy during emotion processing in first-degree relatives of individuals with schizophrenia: a network control study. Published online April 23, 2021:2021.04.22.440935. doi:10.1101/2021.04.22.440935
36. Spilka MJ, Goghari VM. Similar patterns of brain activation abnormalities during emotional and non-emotional judgments of faces in a schizophrenia family study. *Neuropsychologia*. 2017;96:164-174. doi:10.1016/j.neuropsychologia.2017.01.014
37. Villarreal MF, Drucaroff LJ, Goldschmidt MG, et al. Pattern of brain activation during social cognitive tasks is related to social competence in siblings discordant for schizophrenia. *J Psychiatr Res*. 2014;56:120-129. doi:10.1016/j.jpsychires.2014.05.011
38. Dugré JR, Bitar N, Dumais A, Potvin S. Limbic Hyperactivity in Response to Emotionally Neutral Stimuli in Schizophrenia: A Neuroimaging Meta-Analysis of the Hypervigilant Mind. *Am J Psychiatry*. 2019;176(12):1021-1029. doi:10.1176/appi.ajp.2019.19030247
39. Kozhuharova P, Saviola F, Ettinger U, Allen P. Neural correlates of social cognition in populations at risk of psychosis: A systematic review. *Neurosci Biobehav Rev*. 2020;108:94-111. doi:10.1016/j.neubiorev.2019.10.010
40. Saarinen AIL, Huhtaniska S, Pudas J, et al. Structural and functional alterations in the brain gray matter among first-degree relatives of schizophrenia patients: A multimodal meta-analysis of fMRI and VBM studies. *Schizophr Res*. 2020;216:14-23. doi:10.1016/j.schres.2019.12.023
41. Scognamiglio C, Houenou J. A meta-analysis of fMRI studies in healthy relatives of patients with schizophrenia. *Aust N Z J Psychiatry*. 2014;48(10):907-916. doi:10.1177/0004867414540753
42. Luna LP, Radua J, Fortea L, et al. A systematic review and meta-analysis of structural and functional brain alterations in individuals with genetic and clinical high-risk for psychosis and bipolar disorder. *Prog Neuropsychopharmacol Biol Psychiatry*. 2022;117:110540. doi:10.1016/j.pnpbp.2022.110540
43. Fiorito AM, Aleman A, Blasi G, et al. Are Brain Responses to Emotion a Reliable Endophenotype of Schizophrenia? An Image-based fMRI Meta-analysis. *Biol Psychiatry*. Published online June 22, 2022. doi:10.1016/j.biopsych.2022.06.013

44. Brüne M, Ozgürdal S, Ansorge N, et al. An fMRI study of “theory of mind” in at-risk states of psychosis: comparison with manifest schizophrenia and healthy controls. *NeuroImage*. 2011;55(1):329-337. doi:10.1016/j.neuroimage.2010.12.018
45. Abé C, Petrovic P, Ossler W, et al. Genetic risk for bipolar disorder and schizophrenia predicts structure and function of the ventromedial prefrontal cortex. *J Psychiatry Neurosci JPN*. 2021;46(4):E441-E450. doi:10.1503/jpn.200165
46. Zhang Y, Li M, Zhang X, et al. Unsuppressed Striatal Activity and Genetic Risk for Schizophrenia Associated With Individual Cognitive Performance Under Social Competition. *Schizophr Bull*. 2022;48(3):599-608. doi:10.1093/schbul/sbac010
47. Liang C, Pearlson G, Bustillo J, et al. Psychotic Symptom, Mood, and Cognition-associated Multimodal MRI Reveal Shared Links to the Salience Network Within the Psychosis Spectrum Disorders. *Schizophr Bull*. 2023;49(1):172-184. doi:10.1093/schbul/sbac158
48. Madeira N, Martins R, Valente Duarte J, Costa G, Macedo A, Castelo-Branco M. A fundamental distinction in early neural processing of implicit social interpretation in schizophrenia and bipolar disorder. *NeuroImage Clin*. 2021;32:102836. doi:10.1016/j.nicl.2021.102836
49. Feola B, McHugo M, Armstrong K, et al. BNST and amygdala connectivity are altered during threat anticipation in schizophrenia. *Behav Brain Res*. 2021;412:113428. doi:10.1016/j.bbr.2021.113428
50. Hart SJ, Shaffer JJ, Bizzell J, et al. Measurement of Fronto-limbic Activity Using an Emotional Oddball Task in Children with Familial High Risk for Schizophrenia. *J Vis Exp JoVE*. 2015;(106). doi:10.3791/51484
51. Albajes-Eizagirre A, Solanes A, Vieta E, Radua J. Voxel-based meta-analysis via permutation of subject images (PSI): Theory and implementation for SDM. *NeuroImage*. 2019;186:174-184. doi:10.1016/j.neuroimage.2018.10.077
52. Radua J, Mataix-Cols D, Phillips ML, et al. A new meta-analytic method for neuroimaging studies that combines reported peak coordinates and statistical parametric maps. *Eur Psychiatry*. 2012;27(8):605-611. doi:10.1016/j.eurpsy.2011.04.001
53. Zheng D, Xia W, Yi ZQ, et al. Alterations of brain local functional connectivity in amnestic mild cognitive impairment. *Transl Neurodegener*. 2018;7(1):26. doi:10.1186/s40035-018-0134-8
54. Diwadkar VA, Wadehra S, Pruitt P, et al. Disordered corticolimbic interactions during affective processing in children and adolescents at risk for schizophrenia revealed by functional magnetic resonance imaging and dynamic causal modeling. *Arch Gen Psychiatry*. 2012;69(3):231-242. doi:10.1001/archgenpsychiatry.2011.1349
55. Oertel V, Kraft D, Alves G, et al. Associative Memory Impairments Are Associated With Functional Alterations Within the Memory Network in Schizophrenia Patients and Their Unaffected First-Degree Relatives: An fMRI Study. *Front Psychiatry*. 2019;10:33. doi:10.3389/fpsyt.2019.00033
56. Park HY, Yun JY, Shin NY, et al. Decreased neural response for facial emotion processing in subjects with high genetic load for schizophrenia. *Prog Neuropsychopharmacol Biol Psychiatry*. 2016;71:90-96. doi:10.1016/j.pnpbp.2016.06.014
57. Pirnia T, Woods RP, Hamilton LS, et al. Hippocampal dysfunction during declarative memory encoding in schizophrenia and effects of genetic liability. *Schizophr Res*. 2015;161(2):357-366. doi:10.1016/j.schres.2014.11.030
58. Quarto T, Paparella I, De Tullio D, et al. Familial Risk and a Genome-Wide

Supported DRD2 Variant for Schizophrenia Predict Lateral Prefrontal-Amygdala Effective Connectivity During Emotion Processing. *Schizophr Bull*. 2018;44(4):834-843. doi:10.1093/schbul/sbx128

59. Spilka MJ, Arnold AE, Goghari VM. Functional activation abnormalities during facial emotion perception in schizophrenia patients and nonpsychotic relatives. *Schizophr Res*. 2015;168(1-2):330-337. doi:10.1016/j.schres.2015.07.012
60. Wolf DH, Satterthwaite TD, Loughead J, et al. Amygdala abnormalities in first-degree relatives of individuals with schizophrenia unmasked by benzodiazepine challenge. *Psychopharmacology (Berl)*. 2011;218(3):503-512. doi:10.1007/s00213-011-2348-7
61. Hammers A, Allom R, Koepp MJ, et al. Three-dimensional maximum probability atlas of the human brain, with particular reference to the temporal lobe. *Hum Brain Mapp*. 2003;19(4):224-247. doi:10.1002/hbm.10123
62. Tian Y, Margulies DS, Breakspear M, Zalesky A. Topographic organization of the human subcortex unveiled with functional connectivity gradients. *Nat Neurosci*. 2020;23(11):1421-1432. doi:10.1038/s41593-020-00711-6
63. Allen EA, Erhardt EB, Calhoun VD. Data visualization in the neurosciences: overcoming the curse of dimensionality. *Neuron*. 2012;74(4):603-608.
64. Zandbelt, Bram. Slice Display. figshare. Published online 2017. 10.6084/m9.figshare.4742866
65. Guyatt GH, Oxman AD, Kunz R, et al. GRADE guidelines: 7. Rating the quality of evidence—inconsistency. *J Clin Epidemiol*. 2011;64(12):1294-1302. doi:10.1016/j.jclinepi.2011.03.017
